# Occupational Burden of Chronic Obstructive Pulmonary Disease in the Commonwealth of Independent States: Systematic Review and Meta-Analysis

**DOI:** 10.3389/fmed.2020.614827

**Published:** 2021-01-18

**Authors:** Denis Vinnikov, Tatsyana Rybina, Leonid Strizhakov, Sergey Babanov, Irina Mukatova

**Affiliations:** ^1^Environmental and Occupational Health Lab, Al-Farabi Kazakh National University, Almaty, Kazakhstan; ^2^Department of Biochemistry, Peoples' Friendship University of Russia (RUDN University), Moscow, Russia; ^3^Biological Institute, National Research Tomsk State University, Tomsk, Russia; ^4^Scientific and Practical Center MedEvery LLC, Minsk, Belarus; ^5^Department of Internal Medicine, Occupational Diseases and Rheumatology, Sechenov First Moscow State Medical University, Moscow, Russia; ^6^Department of Occupational Diseases and Clinical Pharmacology, Samara State Medical University, Samara, Russia; ^7^Department of Internal Medicine, Nephrology, Hematology, Immunology, and Allergy, Astana Medical University, Nur-Sultan, Kazakhstan

**Keywords:** chronic objective pulmonary disease, dust, occupational, burden, risk

## Abstract

**Background:** Population-based studies from the Russian Federation and neighboring countries on the occupational burden of chronic obstructive pulmonary disease (COPD) are seldom or not included in the systematic reviews. The aim of this review was to summarize published population-based studies from the Commonwealth of Independent States (CIS) in order to ascertain the occupational burden of COPD.

**Methods:** We systematically searched www.elibrary.ru and PubMed for population-based studies on the epidemiology of COPD in nine countries using PRISMA. Quality of studies was assessed using the original tool. The odds of COPD in the included studies from vapors, gases, dusts, and fumes (VGDF) was pooled using meta-analysis (fixed effects model), whereas the population attributable fraction percent (PAF%) was pooled with meta-proportion using the random effects model in Stata 14.2.

**Results:** Five studies, three from Russia, one from Kazakhstan, and one more from Azerbaijan and Kazakhstan (total *N* = 18,908) with moderate to high quality and published from 2014 to 2019 (none from Armenia, Belarus, Kyrgyzstan, Moldova, Tajikistan, and Uzbekistan), were included. Spirometry-defined COPD was the outcome in four of them. The pooled odds ratio (OR) of COPD from VGDF was 1.69 [95% confidence interval (CI) 1.34;2.13], greater in Kazakhstan [OR 1.96 (95% CI 1.35;2.85, *N* = 2 studies)] compared to Russia [OR 1.52 (95% CI 1.13;2.05, *N* = 2 studies)]. The pooled PAF% was 6% (95% CI 2; 14%) from three studies.

**Conclusions:** Population-based studies in the CIS get little attention with very few studies published. Although the effect was greater in Kazakhstan compared to Russia, the overall effect did not differ from studies published in the rest of the world. Research capacity to study occupational risks of COPD should be strengthened to produce more evidence of higher quality.

## Introduction

Chronic obstructive pulmonary disease (COPD) is, by definition, a chronic, usually progressing condition with a global prevalence of 10.7% [95% confidence interval (CI) 7.3;14.0] in people 30 years old and older (1) and is mostly associated with smoking, exposure to secondhand smoke, and vapors, gases, dusts and fumes (VGDF) in the workplace. Recent ERS/ATS statement (2) estimated that the pooled population attributable fraction percent (PAF%) of COPD due to occupational exposures may be as high as 14%, greater in never-smokers (31%). Among occupational exposures associated with COPD, dusty workplaces take the lead; however, particles' aerodynamic diameter (3), mode of exposure, and duration of exposure may be important. Occupational COPD takes years or decades to develop, whereas the individual occupational history and exposure pattern will determine the risk.

The ERS/ATS statement along with preceding reviews mostly considered evidence from the Western countries, since the search of literature was limited to resources in English (1). Such limitation is usually stated in many similar reviews, but no systematic reviews of available literature from the databases in Russian have ever been published. Nine members of the Commonwealth of Independent States (CIS), including Azerbaijan, Armenia, Belarus, Kazakhstan, Kyrgyzstan, Moldova, the Russian Federation (Russia), Tajikistan, and Uzbekistan, occupy 15.6% of the entire land with 3.1% of the entire population on Earth. These countries are known to have had high level of industrialization prior to recent transition period with high ratio of population exposed to VGDF in the industry. In addition, current levels of air pollution in these countries are worrisome (4, 5), adding to high expected prevalence of COPD, but no studies from the region have been systematically summarized in the English literature.

The effect of exposure in the workplace on COPD from these countries remains largely unknown to a reader elsewhere in the globe because of no access to Russian databases. We, therefore, aimed to summarize published population-based studies from these nine countries in order to ascertain the occupational burden of COPD.

## Materials and Methods

### Search Strategy

The primary database to search population-based studies in three selected countries was “Russian index of scientific citations', hosted on www.elibrary.ru. This is a search engine in which most journals published in the Russian Federation and other countries of the former Soviet Union are indexed. Most items returned by this search engine are in Russian language, but few studies in other languages are also present. At www.elibrary.ru, we have elected the broadest search strategy in order to capture all available studies on COPD. We used “обструктивная И население” (obstructive AND population) syntax for all countries, and such keyword combination returned 264 items. The combination “обструктивная И популяционное” [obstructive AND population (as adjective)] identified 45 items; “обструктивная И профессиональная” (obstructive AND occupational) – 235 items; “обструктивная И эпидемиологическое” (obstructive AND epidemiological) – 120 items; “обструктивная И поперечное” (obstructive AND cross-sectional) – 44 items; “обструктивная И когортное” (obstructive AND cohort) – 73 items. Almost 99% of returned items were in Russian language. Finally, we included studies from the inception of the database till November 2020.

Additionally, we performed a similar search in PubMed, but using a more conservative approach in syntax. We used “chronic obstructive pulmonary disease[MeSH Terms] AND (Azerbaijan[MeSH Terms] OR Armenia[MeSH Terms] OR Belarus[MeSH Terms] OR Kazakhstan[MeSH Terms] OR Kyrgyzstan [MeSH Terms] OR Moldova[MeSH Terms] OR Russian Federation[MeSH Terms] OR Tajikistan[MeSH Terms] OR Uzbekistan[MeSH Terms])” to identify all studies from these nine countries. The primary search strategy has been peer-reviewed by an external expert and amended according to PRESS. The final approved search strategy in PubMed returned 141 items.

**Figure 1:**
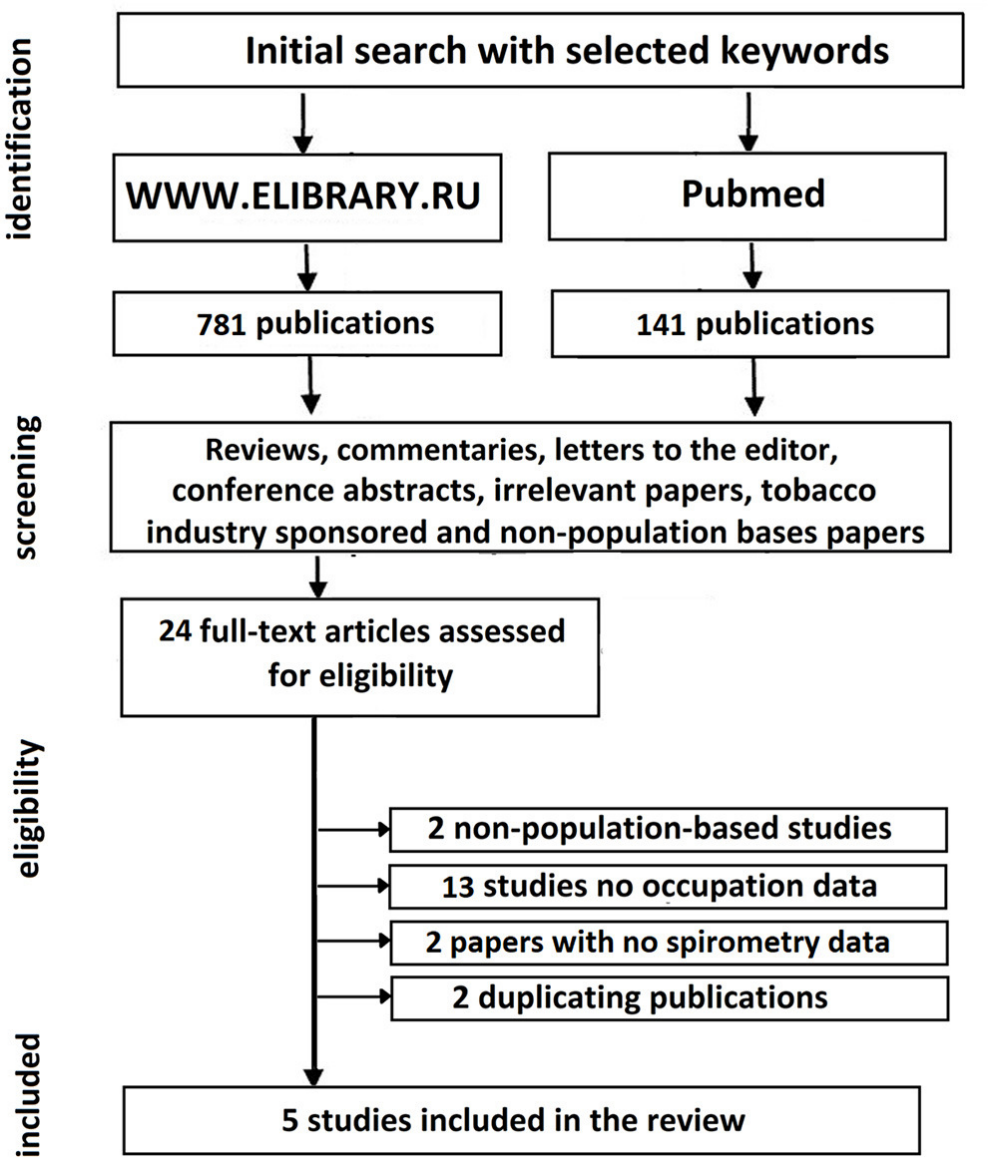
Search strategy and studies selection.

The search in PubMed was accomplished by DV, whereas TR and IM were responsible for studies identification in www.elibrary.ru. For search and data extraction, we followed PRISMA protocol (6), and the protocol checklist accompanies this report as a Supplementary Table. The search strategy along with the subsequent steps of data selection and inclusion can be found in Figure 1.

### Study Selection, Data Extraction, and Assessment of Risk of Bias

Study selection, the subsequent data extraction and quality assessment were done independently by three reviewers, whereas, any disagreements were resolved with a discussion until consensus was reached. During the initial screening of titles and abstracts at www.elibrary.ru, we excluded conference presentations, reviews, letters to the editor, irrelevant reports on any other topics not related to COPD epidemiology, such as treatment, as well as all studies not showing clear evidence of being done in the general population, including studies in the workers of plants, factories, and other selected groups. Of note, most returned studies from www.elibrary.ru were carried out in such occupational groups. Moreover, we had to exclude all studies published in the so-called “Chernobyl nuclear station liquidators,” despite some dust exposure reported in some of them. Chemical composition of that dust has clearly had radioactive components, resulting in significant comorbidity, whereas the duration of employment at that disaster site was not long enough to cause COPD compared to other occupations. In addition, we also excluded studies and study protocols sponsored by the tobacco industry. We screened references in the identified papers in order to find reports that might have been omitted using the selected search strategy.

Once the full-text papers found in PubMed were independently read by three authors, we excluded one study, in which spirometry was not done; one non-population-based study; four population-based studies where occupational exposures were not assessed nor eventually reported; and one study where only COPD patients were included, although the abstract stated that the study was population-based. From the pool of eligible studies found at www.elibrary.ru and published in Russian, we excluded nine studies because they did not assess occupational risk factors or provided very little information on them, not allowing data extraction; one study where only younger patients (18 to 44 years) were enrolled; and two papers that duplicated reports earlier published by the same team in English. The summary of inclusion and exclusion criteria is presented in Table 1.

**Table 1:** Inclusion and exclusion criteria for studies in the current analysis.

**Inclusion criteria**	**Exclusion criteria**
Population-based studies Studies where COPD was defined with spirometry Studies with clear definition of exposure	Conference presentations Reviews Letters to the Editor Studies in selected populations (occupational cohorts) Studies in Chernobyl nuclear station liquidators Studies with unclear exposure data Studies in young population only Studies where the diagnosis was not confirmed with spirometry Duplicate reports Studies sponsored by the tobacco industry

From each study, we extracted data on the first author and year, country, location, sample size and age distribution, detailed description of exposure and exposure assessment methods, outcome and its assessment, magnitude of populational exposure, i.e., number of people exposed in the entire sample, and the effect measure, such as the odds ratios (ORs) with their corresponding 95% CI. Whenever the latter were not reported, we extracted the number of people with and without the exposure in COPD and non-COPD groups. We also looked for PAF% in each included study. PAF% is the proportional reduction in population disease or mortality that would occur if exposure to a risk factor were reduced to an alternative ideal exposure scenario. Whenever that was not reported, we contacted the authors twice via e-mail to obtain missing data, such as proportion of subjects exposed needed for PAF% computation.

Because general quality assessment tools may not fully capture important aspects of methodology specific for epidemiological studies of COPD, we have worked out our own set of a quality assessment score. The score is calculated as a sum of scores, each corresponding to one of the answers in a set of multiple-choice questions. We included specific questions on biases, study design, sample size, and the overall presentation clarity, pertinent for epidemiological studies of COPD. These criteria have been worked out based on a literature review of earlier population-based studies of COPD. The items, possible answers, and their corresponding scores are presented in Table 2. The scores from each question for each study were summed, producing the overall score from 0 to 17. Studies with the overall score from 0 to 4 were considered of very low quality; 5 to 8—low quality; 9 to 12—moderate quality; and 13 to 17—high quality. Such grading is simply based on the quartiles of the overall score from 0 to 17 and correspond to quartiles 1, 2, 3, and 4.

**Table 2:** Questions along with answers and their corresponding scores for quality assessment.

**Question**		**Answers**		
1.	Has the study been designed solely for COPD?	Yes (1)	No or Unclear (0)	
2.	How large was the sample?	More than 2000 (2)	501–2000 (1)	0–500 (0)
**Selection bias**				
3.	Is there clear evidence that subjects were randomly selected from the generation population?	Yes (2)	Unclear (1)	No (0)
4.	Did the sample include adults of all ages?	Yes (1)	No or Unclear (0)	
5.	Was the study done in only one city?	No (1)	Yes or Unclear (0)	
**Classification bias**				
6.	Was there any instrumental verification of exposure?	Yes (1)	No or Unclear (0)	
7.	Were dust and/or vapors, gases, fumes clearly stated as an exposure?	Yes (1)	No or Unclear (0)	
8.	Were the questionnaires built on the international, validated tools?	Yes (1)	No or Unclear (0)	
9.	Did the authors use job exposure matrix (JEM) rather than collecting individual work history?	No (1)	Yes (0)	
10.	Was spirometry done in all subjects?	Yes (2)	Unclear (1)	No (0)
11.	Did the authors consider patients to have COPD only after bronchodilation?	Yes (1)	No or Unclear (0)	
12.	Were ERS/ATS guidelines of spirometry quality considered?	Yes (1)	No or Unclear (0)	
**Confounding**				
13.	Were the models adjusted for significant confounders?	Yes (1)	No or Unclear (0)	
14.	Did the models adjust for smoking?	Yes (1)	No or Unclear (0)	

### Data Synthesis and Analysis

The primary endpoint in this analysis was the OR with its corresponding 95% CI calculated in either crude or adjusted for confounders analyses in each included study. This represented the odds of developing COPD in those exposed to VGDF or only dusts when compared to those who did not report such exposure. In those studies where ORs were not presented, we extracted data on the number of exposed vs. non-exposed participants separately in COPD and non-COPD groups and constructed contingency tables, which yielded crude ORs. Whenever adjusted regression models were reported, we extracted data on a list of specific confounders in a given study. In a meta-analytic procedure, we pooled ORs from each included study using fixed effects models considering low heterogeneity between studies. The latter was first calculated and reported with *I*^2^ and its corresponding *p* value. Fixed effect model calculates weight of each individual study considering precision only, a function of a sample size. We also report stratified meta-analyses of groups of countries, in which at least two studies could be pooled (the Russian Federation vs. Kazakhstan).

Because heterogeneity between studies was low, we did not perform sensitivity analysis. Publication bias was tested using Begg's and Egger's tests. Crude ORs were calculated manually. In addition, we also pooled published PAF% from the studies using meta-proportion function. Whenever PAF% was not reported, we calculated it using the formula: $\text{PAF}\%\text{=}\frac{{\text{p}}^{*}(\text{OR-}1)}{{\text{p}}^{*}\left(\text{OR-}1\right)\text{+}1}$. Such computation was only possible in studies that reported OR and the percentage of exposed population or responded to our e-mail enquiry asking to provide the information needed. Exact method was used to calculate 95% CIs for the PAF% estimates. Given high heterogeneity between studies in PAF%, we report random effect model pooled PAF% from the meta-proportion function with Freeman–Tukey transformation to stabilize the variances. Meta-analysis and meta-proportion were performed in Stata 14.2 (Texas, USA).

## Results

Twelve studies were selected for full-text extraction from www.elibrary.ru along with 12 studies to be downloaded as full-text articles to screen eligibility for inclusion from PubMed. Eligibility screening of the full texts of articles from PubMed allowed us to narrow the number of eligible studies to five, which were published between 2014 and 2019 (7–11). The study of Nugmanova et al. was conducted in three countries, including Azerbaijan, Kazakhstan, and Ukraine; therefore, data from Azerbaijan and Kazakhstan could be analyzed in the current presentation and were extracted separately. From the pool of 12 eligible studies identified at www.elibrary.ru, none were included in the final analysis.

### Russia

Our search yielded three population-based studies published between 2014 and 2019, which we could include in the current analysis. The overall sample was 15,530 subjects from 12 major cities (7), Bashkortostan (10), and Saint-Petersburg with Arkhangelsk (8). The earliest presentation of Chuchalin et al. (7) with the largest sample of all included studies was from 12 major cities, accomplished as part of GARD project in Russia (Table 3). Along with other predictors, dusty job for at least 1 year was defined as an exposure of interest for the current analysis, whereas spirometry was only done in a small group (16%) of subjects with symptoms. Since COPD was confirmed with spirometry only in a small group of those with symptoms, here we report the number of subjects with self-reported chronic bronchitis. Therefore, the outcome extracted for this analysis was self-reported ever-diagnosis of chronic bronchitis. In an unadjusted analysis, dusty job increased the likelihood of the outcome 2.5-fold (Table 3).

**Table 3:** Summary table of the studies included in the review.

**#**	**References**	**Country**	**Region**	**Sample**	**Exposure**	**Exposure assessment**	**Outcome**	**Outcome assessment**	**% exposed**	**Effect with 95% CI**
1	Chuchalin et al. (7)	Russian Federation	12 major cities	7164	Dusty job, at least 1 year	Questionnaire	Chronic bronchitis	Symptoms	22%	OR 2.58 (2.17;3.08), crude
2	Andreeva et al. (8)	Russian Federation	Northwest	2974	Dusty job, at least 1 year	Questionnaire	FEV_1_/FVC below GLI LLN	Spirometry	13%	OR 1.14 (0.66;1.87), adjusted for age, sex, smoking, exposure to gas, chemicals, biomass, chronic cough, and dyspnea
3	Nugmanova et al. (9)	Azerbaijan and Kazakhstan	Baku and Almaty	933 (Baku) and 945 (Almaty)	Dusty work	Questionnaire	Postbronchodilator FEV_1_/FVC < 0.7	Spirometry	Unspecified	OR 1.70 (0.80;3.60) in Baku;OR 2.31 (1.33;4.00) in Almaty, crude
4	Bilbov et al. (10)	Russian Federation	Baskhortostan	5392	Dusty work	Questionnaire	FEV_1_/FVC below GLI LLN	Spirometry	7%	OR 1.75 (1.22;2.50), crude*
5	Vinnikov et al. (11)	Kazakhstan	Almaty	1500	All positions in a lifetime held recorded, cumulative work duration for VGDF jobs calculated	Questionnaire	Postbronchodilator FEV_1_/FVC < LLN	Spirometry	26%	OR 1.71 (1.03;2.84), adjusted for age, sex, smoking, income and exercise

*ORs and their 95% CIs were calculated by the authors of this review from the % exposed and unexposed in COPD and non-COPD groups provided in the tables of the manuscript; FEV_1_, forced expiratory flow in 1 s; FVC, forced vital capacity; LLN, lower limit of normality; GLI, Global Lung Function Initiative; VGDF, vapors, gases, dusts, and fumes; CI, confidence interval.

In 2016, Andreeva et al. (8) published the findings of a population-based study, which was completed in two cities of Russian Northwest, including Saint Petersburg and Arkhangelsk. Randomly selected population (*N* = 2974) from 15 primary care centers aged 35–70 years was included in the analysis, whereas spirometry tests with bronchodilation were available in 2,388 participants. Dusty job for at least 1 year was the exposure of interest, and adjusted regression models were used for two outcomes, including FEV_1_/FVC < 0.7 fixed ratio or FEV_1_/FVC below lower limit of normal (LLN) obtained from Global Lung Function Initiative (GLI) reference values. For those who were exposed to dusty jobs for more than 10 years, the OR of FEV_1_/FVC < 0.7 adjusted for age, sex, smoking, exposure to gas, chemicals, biomass, chronic cough, and dyspnea, was 1.05 (95% CI 0.67;1.64) and, alternatively 1.14 (95% CI 0.66;1.87) for FEV_1_/FVC below LLN. In all multivariate regression models, including those with fewer predictors, the association was non-significant.

In the study of Bilbov et al. (10), respiratory health was assessed as part of Ural Eye and Medical Study in Bashhkortostan. The analysis was built on 5,392 subjects 40 years and older, who underwent spirometry and it showed good quality. The questionnaire, above all, comprised questions on the occupational dusts. Airway obstruction was defined as FEV_1_/FVC below LLN. No regression data are reported by the authors; however, the authors state that the airway obstruction group (5.8%) had “higher occurrence rate of dust at the working place.”

### Kazakhstan

There were no population-based studies on the association of occupational risk factors with COPD prior to 2018 from Kazakhstan. Two recently published reports had the overall sample of 2,445 subjects and were both carried out in Almaty, the largest city with the population of about 2 million. Despite large territory, no reports from other cities, including the capital, Nur-Sultan (for Astana), could be included in this report because they were not yet published. Earlier study of Nugmanova et al. (9) was accomplished in three countries of the former Soviet Union, Kazakhstan, Ukraine, and Azerbaijan using similar methodology and a cluster randomization approach. In Kazakhstan, they recruited 945 subjects in Almaty, visiting their places of residence and performing all tests, including spirometry with bronchodilation, at home. COPD was confirmed in those with postbronchodilator FEV_1_/FVC < 0.7. No detailed occupational history was collected, but those ever working in dusty jobs were considered an exposed population. The crude OR of COPD of such exposure was 2.31 (95% CI 1.33;4.00).

Alternatively, in the recent study of Vinnikov et al. (11), also conducted in Almaty, lifetime occupational history was collected. Moreover, COPD was confirmed in subjects having postbronchodilator FEV_1_/FVC < LLN, and the association was tested in multivariate regression models adjusted for age, sex, smoking, income, and exercise. A number of ever held occupations were considered as VGDF jobs, and known work duration allowed for stratification into those exposed for 0–9, 10–22, and 23+ years. The overall adjusted OR of COPD was 1.71 (95% CI 1.03;2.84), increasing to 2.36 (95% CI 1.20;4.66) in those classified as having 23+ years of exposure.

### Azerbaijan

In a population-based study of Nugmanova et al. (9), they recruited 933 subjects in Baku, the capital of Azerbaijan. As stated above, COPD was verified using spirometry in patients with postbronchodilator FEV_1_/FVC < 0.7. No detailed occupational history of subjects was reported, but those stating they ever worked in dusty jobs had a non-significant greater chance of having COPD [OR 1.7 (95% CI 0.8;3.6)]. No adjusted effects of occupational exposures are available from this report.

### Armenia, Belarus, Kyrgyzstan, Moldova, Tajikistan, and Uzbekistan

No studies on the epidemiology of COPD with data on occupational exposures, which could be eligible for inclusion in the current presentation, were identified in Armenia, Belarus, Kyrgyzstan, Moldova, Tajikistan, and Uzbekistan.

### Quality Assessment of Included Studies

Table 4 shows that the overall score related to quality control ranged from 10 to 15, assuming there were no studies of very low or low quality. Two studies out of five included reports that were classified as having high quality. In all studies, there was a clear evidence that subjects were randomly selected from the general population, and only two out of five studies were confined to one city. Exposure was classified with questionnaires only in all included reports. Moreover, in 80% of included publications, dust was clearly pronounced as an exposure of interest. Although smoking as a variable was reported in the papers, regression models were adjusted for it in only two (40%) of them. Since smoking may be a significant confounder in all occupational studies, especially in COPD, studies with the effect adjusted for smoking will provide more precise data and get a higher score.

**Table 4:** Quality assessment grading of included studies.

**Item**	**Chuchalin et al. (7)**	**Andreeva et al. (8)**	**Nugmanova et al. (9)**	**Bilbov et al. (10)**	**Vinnikov et al. (11)**
1	1	1	1	0	1
2	2	2	1	2	1
3	2	2	2	2	2
4	1	0	1	0	1
5	1	1	0	1	0
6	0	0	0	0	0
7	0	1	1	1	1
8	1	1	1	0	1
9	1	1	1	1	1
10	0	2	2	2	2
11	1	1	1	0	1
12	1	1	1	1	1
13	0	1	0	0	1
14	0	1	0	0	1
Total score	11	15	12	10	14
Grading	Moderate	High	Moderate	Moderate	High

### Meta-Analysis

**Figure 2:**
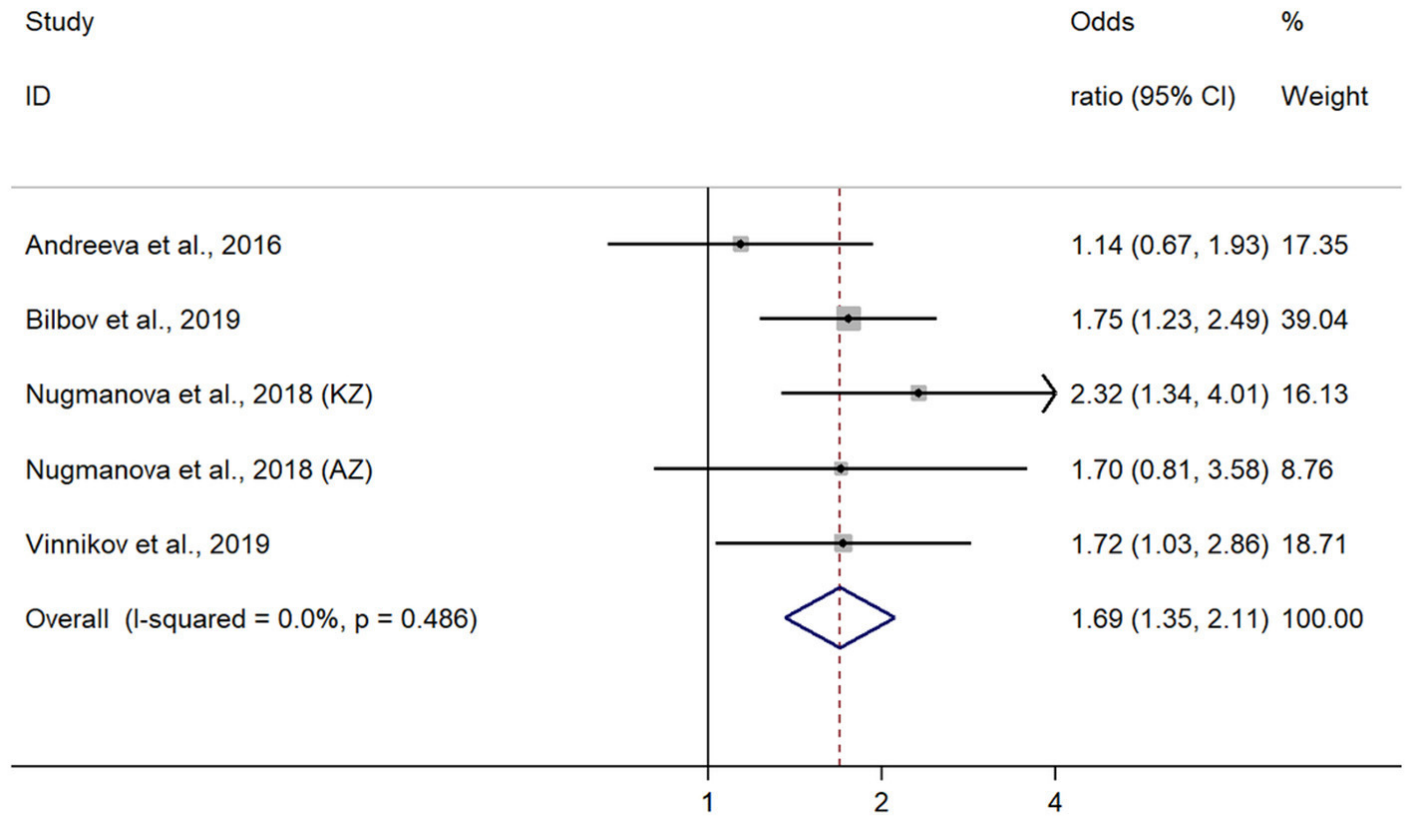
Forrest plot on four included studies with their effects.

**Figure 3:**
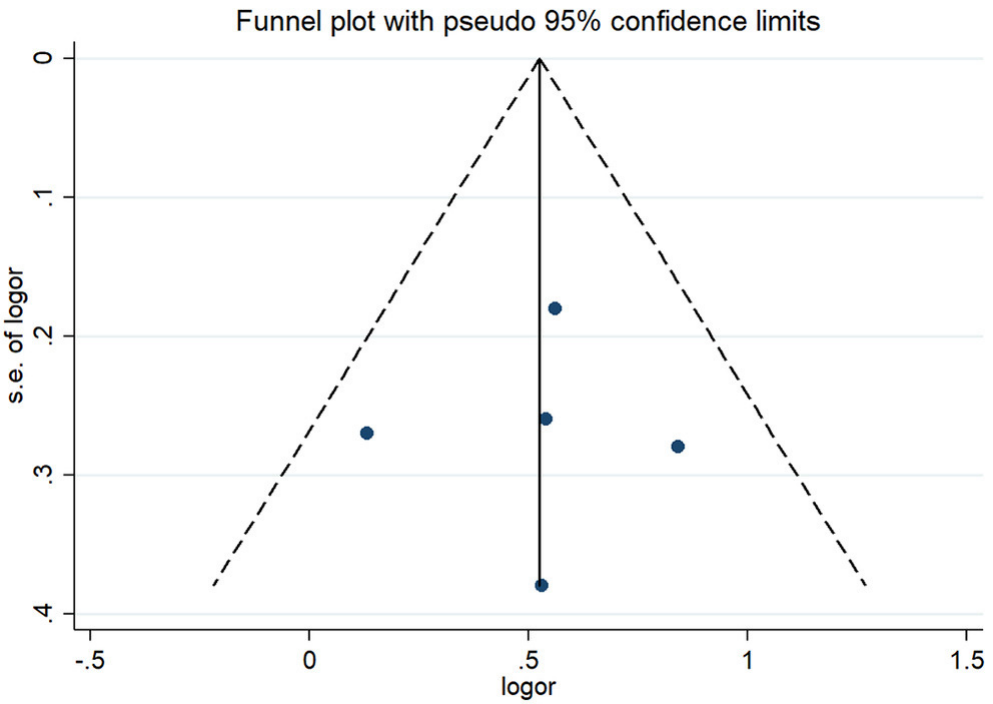
Funnel plot of included studies showing no publication bias.

Given that all included publications were of moderate or high quality, consistent in the effect measure reported, we considered conducting meta-analysis in order to produce the pooled effect. The pooled OR of COPD in five studies from three countries (Azerbaijan, Kazakhstan, and Russia) was 1.90 (95% CI 1.45; 2.48) using the random effects model with high heterogeneity across studies (*I*^2^ = 59%, *p* = 0.03). Because the study of Chuchalin et al. reported COPD verified with spirometry only in a small group of subjects, we ran subgroup analysis of four studies, where spirometry was used to confirm the diagnosis in all subjects. In the pooled analysis of a subgroup of four included studies from three countries, the effect was homogeneous (*I*^2^ = 0%, *p* = 0.49) across the studies using the fixed effects model. In such analysis, where the weight of studies is calculated solely based on sample size, the study of Bilbov et al. had the greatest weight in the final effect (39%). In this subgroup, the pooled OR of COPD in those exposed to dust or VGDF was 1.69 (95% 1.34;2.11) from four studies, including two in Russia, one in Kazakhstan, and the remaining one in Azerbaijan and Kazakhstan (Figure 2). When studies were stratified in those conducted in the Russian Federation (*N* = 2) and those in Kazakhstan (*N* = 2), we observed a greater effect in Kazakhstan studies, although in the similar direction of effect. Thus, the pooled OR of studies from the Russian Federation was 1.52 (95% CI 1.13;2.05), whereas two studies from Kazakhstan yielded the OR 1.96 (95% CI 1.35;2.85). In addition, Egger's and Begg's tests showed no evidence of publication bias. The corresponding *p* values for these tests were 0.93 and 0.81. The funnel plot of included studies did not show publication bias as well (Figure 3).

We had the needed data to run meta-proportion of PAF% for three studies only. The study of Chuchalin et al. was excluded from the analysis for the reasons mentioned above, whereas the study of Nugmanova et al. could not provide percentage of exposed population. In a meta-proportion analysis, heterogeneity between studies was very high (*I*^2^ = 99%, *p* < 0.001); therefore, we applied the random effects model for this test. The pooled PAF% for three included studies [Andreeva et al. (8), 1.78%; Bilbov et al. (10), 4.99%; and Vinnikov et al. (11), 15.6%] was 6% (95% CI 2;14). The source of such heterogeneity was the study of Vinnikov et al. from Kazakhstan, in which PAF% was significantly different from the Russian studies, because it was a specially designed study with a detailed occupational history analysis. In contrast, two Russian studies were designed to verify COPD prevalence and one question on occupational history was among dozens of other risk factors in the questionnaire. In a stratified analysis, two studies from the Russian Federation yielded the pooled PAF% 4% (95% CI 3;4).

### Synthesis

The number of studies providing the evidence in accordance with the inclusion criteria was too low for a large territory of nine countries of interest. Among these, eligible studies were published only from the Russian Federation, Kazakhstan, and Azerbaijan. All included studies were consistent in both the direction and magnitude of effect. Occupational exposure to VGDF in these countries increased the likelihood of COPD almost twofold, but the effect was lower in studies adjusting for confounders. The overall quality of evidence from included studies was moderate to high.

## Discussion

This is the first report on the systematic search of population-based studies from nine countries of the former Soviet Union, now part of the CIS, including Azerbaijan, Armenia, Belarus, Kazakhstan, Kyrgyzstan, Moldova, Russia, Tajikistan, and Uzbekistan. The search of studies was completed in the leading Russian medical database, which indexed almost all scientific journals published in Russian language, supplemented by a similar search in PubMed. We found only five reports of studies with the design of interest, indicative of insufficient interest to obtaining local evidence on the risks for COPD in selected countries. Most studies were published in the last 5 years, reflecting some rise of interest to study the risk of COPD only recently. When four studies with data from three countries were included in meta-analysis, we found that occupational exposures, classified with questionnaires, increased the risk of COPD 1.69-fold.

The Russian Federation traditionally has a very high level of industrialization with likely high exposure of workers to VGDF in multiple sites of metallurgy, machining, metalworks, construction, and other related industries. According to Rosstat (Federal State Statistic Service) (www.gks.ru), 37.9% of those employed in the industry are exposed to occupational hazards (12), more in males (45.4%) compared to females (23.1%). Of all occupational hazards, the fraction of workers with exposure to aerosol, mostly the one with high silica content, is indeed smaller and equals 4.6% (12). In addition, 7.9% of the working population are exposed to chemicals (12). The greatest fraction of those exposed to aerosol is in coal mining (27.4%) and metallurgic production (22.0%). These numbers correspond to aerosol with high silica content only and do not account for other VGDFs. Yet, the number of published reports from Russia remains very low. Unavailability or very poor funds in public health research, including occupational health, can explain such gap, since research in occupational health is not a priority in the governmental research programs. In nine countries included in this review, academic activity in occupational health is traditionally focused in the specialized research institutes rather than in the universities. Their research interests, in turn, are still concentrated in the treatment of already existing diseases as opposed to their prevention and risk assessment. In addition, in Russia and in other neighboring countries, occupational diseases associated with industrial aerosol do not take the lead in the overall occupational morbidity.

Among two countries, for which we could calculate PAF%, the burden of occupational COPD was greater in Kazakhstan, resulting from more self-reported exposed population included in the samples. These numbers, however, do not consider some exposure to particulate matter (PM) in the workplaces, not traditionally considered hazardous, but shown to have high concentrations of pollutants. A recent study in Almaty (5) has demonstrated extremely high levels of PM_10_ in winter in Almaty from fossil fuel burning for heating and cooking either by households in the city and in the suburbs or central heating plants in the city. Such significant exposure has serious implications with regard to whom to consider an exposed population. All those working outdoors in the city in winter may also experience severe health effects of air pollution in the workplace because of high ambient PM concentrations, but they are yet not classified as exposed population. Moreover, they may be unaware of the risks imposed. Taken together, we assume that the true fraction of exposed population in the workplace in Almaty and possibly in other cities of Kazakhstan may be higher, with even greater PAF% than reported.

No population-based studies from highly industrialized Belarus was a surprising finding in this review. In a population of industrial workers in Belarus, few reports have shown that the risk in highly exposed population vs. those with the least exposure was doubled, such as in the study of Minsk Tractor Plant (13). Identification of the reasons why population-based studies from Belarus are not yet published in research goes beyond the scope of the current analysis; however, poor funding of public health research and low interest of universities may explain low priority of studying occupational risks of respiratory diseases in this country. The same relates to Kazakhstan, where two existing studies were published only in 2018–2019, whereas no population-based studies were completed before. Of note, the primary database in this review, www.elibrary.ru, dates back to ~2005, whereas earlier publications only exist in their paper version and cannot be indexed by the electronic database at the moment. Very slow database digitalization hampers the access of research community to earlier studies. In addition, searching for studies in the paper catalogs would require a lot of effort, but the output of such local access to paper databases is doubtful.

Studies from Armenia, Moldova, Kyrgyzstan, Tajikistan, and Uzbekistan either were not published or failed to report occupational risk factors. Thus, in a few publications from Kyrgyzstan, where the effect of altitude and even household air pollution on COPD is analyzed, occupational data are poorly presented. The fact that the search returns zero items in Moldova and Tajikistan is indicative of very little attention to COPD in these countries or, alternatively, of serious lack of scientific infrastructure for population-based studies.

The pooled OR of COPD from exposure to dust in three countries included in this analysis (Azerbaijan, Kazakhstan, and Russian Federation) does not differ from what is known from systematic reviews elsewhere in the West. A bit higher in Kazakhstan studies, the odds of having COPD was almost twice greater in the exposed population. The ORs obtained in other countries using a similar study design were all approximately around 2, whether exposure was assessed via JEM (14, 15) or self-reported (16, 17). The effect was greater in studies with crude analyses, not accounting for smoking. In contrast, pooled PAF% (4%) was surprisingly low from two eligible Russian studies, when compared to published reviews elsewhere (2). The reasons behind such reduced fraction have to be further elucidated, given that the number of workers exposed to occupational hazards as industrial aerosol and chemicals cannot be considered negligible (12). Because PAF% is directly derived from the percentage of population exposed, such low PAF% resulted from quite a small number of exposed subjects in both studies, down to 7%. Given that standardized questionnaires were applied in these studies, low fraction of population with exposure to VGDF can then be explained by the skewness toward less exposed population in the sample. The contract is obvious when comparing studies specifically designed to ascertain the burden of occupational exposures (11) with those where risk factors for COPD were multiple and occupational hazards were not the risks of primary interest (8, 10). Taken together, all that highlights the need for better occupational history collection in the future population-based studies in these countries.

In the current analysis, we found that studies with crude ORs had a greater effect compared to those where the effect was adjusted for smoking. The latter is an important confounder in occupational studies, because exposed population likely has higher rates of smoking; therefore, this variable should be considered in all occupational studies. Of five included studies, only two (40%) adjusted the ORs for smoking, indicative of potentially some bias of the effect away from the null. Although adjusting for smoking will change the effect by ~25% (18), smoking should always be considered in the occupational studies in order to report more accurate association. Should smoking be taken into account in two studies where crude ORs were reported, the overall effect would be lower and closer to numbers reported in two studies with such adjustment.

This study has a number of strengths. First and foremost, this is the only report from the former Soviet Union to systematically analyze the major database in Russian language. Secondly, we combined the search in the Russian database with PubMed, making this presentation the widest summary of published studies from nine included countries ever completed. The limitations should also be listed. Firstly, we limited this search to only nine countries of the former Soviet Union and could not include Baltic countries, Georgia and Ukraine. Secondly, we used our own scale for quality assessment, adapted specifically for COPD, which is both the strength and the limitation of this review. Such approach allowed us to better tackle biases, specific for studies in respiratory medicine. One more limitation is the use of crude (not adjusted for smoking) effects of the occupational exposure for meta-analysis from three out of five included studies, because adjusted effects were not reported. Finally, the number of included studies was too small to apply more stratification or sensitivity analyses to better understand any potential sources of heterogeneity.

In this first report of the systematic search of population-based studies in nine countries of interest, including Azerbaijan, Armenia, Belarus, Kazakhstan, Kyrgyzstan, Moldova, the Russian Federation, Tajikistan, and Uzbekistan on the occupational burden of COPD, we found that studies of such design appeared only since 2014, and their count remains too low to date. Six countries, including Armenia, Belarus, Kyrgyzstan, Moldova, Tajikistan, and Uzbekistan, have never published population-based studies of sufficient quality of exposure and occupational data. For such enormous territories, the evidence of the association of exposure to VGDF with COPD remains insufficient, samples in published studies are quite small, whereas most studies were not designed specifically for COPD. The overall PAF% due to exposure may be underestimated, resulting from fewer exposed population included in these reports. Further high-quality population-based studies are needed to better address and classify exposure in these countries.

## Data Availability Statement

The original contributions presented in the study are included in the article/Supplementary Material, further inquiries can be directed to the corresponding author/s.

## Author Contributions

DV: study design, data collection, data analysis, and manuscript drafting. TR: data collection, data analysis, and final version approval. LS: data analysis and final version approval. SB: data analysis, manuscript drafting, and final version approval. IM: data collection, data analysis, and final version approval. All authors contributed to the article and approved the submitted version.

## Conflict of Interest

TR was employed by the company Scientific and Practical Center MedEvery LLC. The remaining authors declare that the research was conducted in the absence of any commercial or financial relationships that could be construed as a potential conflict of interest.
